# Postponing intubation in spontaneously breathing major trauma patients upon emergency room admission does not impair outcome

**DOI:** 10.1186/s13049-019-0656-9

**Published:** 2019-08-28

**Authors:** Philipp Schwaiger, Herbert Schöchl, Daniel Oberladstätter, Helmut Trimmel, Wolfgang G. Voelckel

**Affiliations:** 1 Departement of Anaesthesiology and Intensive Care Medicine AUVA Trauma Centre Salzburg, Academic Teaching Hospital of the Paracelsus Medical University, Dr.-Franz-Rehrl-Platz 5, Salzburg, Austria; 2 Ludwig Boltzmann Institute for Experimental and Clinical Traumatology, AUVA Trauma Research Centre, Vienna, Austria; 3 OEAMTC Air Rescue, Baumgasse 129, 1030 Vienna, Austria; 4 Department of Anaesthesiology, Emergency and Critical Care Medicine, Wiener Neustadt General Hospital, Corvinusring 3-5, 2700 Wiener Neustadt, Austria; 5 Karl Landsteiner Institute of Emergency Medicine, Corvinusring 3-5, 2700 Wiener Neustadt, Austria; 6 Network for Medical Science, University of Stavanger, Stavanger, Norway

**Keywords:** Airway management, Pre-hospital intubation, Emergency room intubation, Outcome, Major trauma

## Abstract

**Background:**

Pre-hospital emergency anaesthesia and tracheal intubation are life-saving interventions in trauma patients. However, there is evidence suggesting that the risks associated with both procedures outweigh the benefits. Thus, we assessed whether induction of anaesthesia and tracheal intubation of trauma patients can be postponed in spontaneously breathing patients until emergency room (ER) admission without increasing mortality.

**Methods:**

Retrospective analysis of major trauma patients either intubated on-scene by an emergency medical service (EMS) physician (pre-hospital intubation, PHI) or within the first 10 min after admission at a level 1 trauma centre (emergency room intubation, ERI). Data was extracted from the German Trauma Registry, hospital patient data management and electronic clinical information system.

**Results:**

From a total of 946 major trauma cases documented between 2010 and 2017, 294 patients matched the study inclusion criteria. Mortality rate of PHI (*N* = 258) vs. ERI (*N* = 36) patients was 26.4% vs. 16.7% (*p =* 0.3). After exclusion of patients with severe traumatic brain injury and/or pre-hospital cardiac arrest, mortality rate of PHI (*N* = 100) vs. ERI patients (*N* = 29) was 6% vs. 17.2%, (*p* = 0.07). Median on-scene time was significantly (*p* < 0.01) longer in PHI (30 min; IQR: 21–40) vs. ERI patients (20 min; IQR: 15–28).

**Conclusions:**

There was no statistical difference in mortality rates of spontaneously breathing trauma patients intubated on-scene when compared with patients intubated immediately after hospital admission. Due to the retrospective study design and small case number, further studies evaluating the impact of airway management timing in sufficiently breathing trauma patients are warranted.

## Background

It is undisputed that professional airway management, appropriate oxygenation and ventilation save lives when provided as early as possible [1, 2]. On the other side, there is evidence that patients may be put at risk when airway management is inappropriate and performed by non-skilled operators, thus decreasing the likelihood of survival [3, 4]. Recently, several publications raised the question whether airway management is always mandatory in a wide variety of emergencies such as shock, multiple- or brain trauma [5]. For non-traumatic emergencies, data advocating emergency anaesthesia and tracheal intubation is more than spare. A current Cochrane Database review addressing emergency intubation for acutely ill and injured patients states that the efficacy of emergency intubation is still unproven and should be more rigorously studied [6].

In spontaneously breathing patients, emergency physicians must carefully outweigh the risk-benefit ratio of emergency anaesthesia and intubation, and deliberately decide whether airway management should be provided as early as possible in the pre-hospital phase, or postponed until hospital admission. Since current data is inconclusive, we sought to assess whether emergency medical personnel could safely refrain from induction of anaesthesia and intubation of critical, yet spontaneously breathing severe trauma patients, with regard to a higher standard of care that is available within the environment of an emergency room.

Accordingly, we retrospectively analysed patients admitted to our AUVA Level 1 Trauma Centre in Salzburg, Austria that were intubated either in the pre-hospital setting on-scene or during the initial phase of in-hospital care in the emergency room. Our null-hypothesis was that survival rates are comparable between both groups.

## Methods

Retrospective analysis of major trauma patients admitted to the AUVA Level 1 Trauma Centre Salzburg between August 2010 and December 2017 with an Injury Severity Score (ISS) > 9 and the need for > 24 h intensive care treatment. Only patients who have been already intubated in the pre-hospital setting by the emergency medical service (EMS), and patients intubated within the first hour after admission to the emergency room were included and further analysed.

### Setting

Austria in general, and Salzburg in particular relies on a tight net of EMS systems comprising EMT ambulance cars as well as physician-staffed ground and air rescue services. The Salzburg Trauma Network further ensures that patients suffering of major injuries are directly transported to one of the two Level 1 Trauma Centres by either ground ambulance or helicopter emergency service (HEMS). The AUVA Trauma Centre Salzburg is a certified Level I trauma centre, located in downtown Salzburg, Austria. It is one of the two supra-regional centres within the aforementioned Trauma Network. In average, 150 major trauma cases deriving from Salzburg state and surrounding regions are admitted per year. Of all major trauma patients, comprehensive in-hospital data is available, thus allowing an insight in patient care and outcome assessment.

### Data collection

Data was obtained by extracting the Salzburg Trauma Centre patients from the German Trauma Registry Database, and subsequently, analysed together with the data derived from the hospital electronic patient records comprising the electronic patient data management system (PDMS) COPRA 6™ (Berlin, Germany), and the AUVA electronic clinical information system (ASTRA). Furthermore, the electronic radiography picture archiving and communication system (PACS) was employed as needed. All data obtained was handled according to current data protection guidelines as defined in the General Data Protection Regulation (EU-GDPR) allowing the processing of personal data necessary for the purposes of management of health care systems (Art. 9.2). The obligations for all persons involved in data processing are defined within our institution by the data protection officer and formally acknowledged. Data were collected anonymously in an MS Excel sheet (Microsoft, Redmond, WA, USA) and stored on data protected institutional hardware.

### Case by case analysis and categorization of indications for intubation

The data provided by the Trauma Registry comprised patients who were intubated on-scene by emergency physicians and those who were intubated in the emergency room by the receiving trauma team. The latter group of patients was anaesthetized due to acute, vital indications that were present upon emergency room admission, or intubated because of other reasons. In order to differentiate between vital, urgent or delayed airway management, a case-by-case analysis of all emergency room intubations was conducted and four major groups were defined (Table 1). For comparisons, only group 1 indications comprising patients with an acute, vital indication for emergency anaesthesia and intubation were included.

**Table 1:** Indications for emergency anaesthesia and intubation in the emergency room

Group	Indication
1	Acute vital indication existing upon hospital admission. The indication was already given during the pre-hospital phase.
2	Acute vital indication emerging during the emergency room phase (eg, deteriorating patient). The indication did not exist during the pre-hospital phase.
3	Anaesthesia and intubation due to a combative/agitated patient or for the purpose of analgesia and/or diagnostic interventions.
4	Anaesthesia and intubation needed for pursuing further treatment following CT-diagnostics and achievement of interdisciplinary consensus (eg, immediate surgery or emergency room interventions under general anaesthesia).

### Scoring systems

Besides the Glasgow Coma Scale (GCS) derived from the pre-hospital EMS and primary emergency room assessment, three other scoring systems were employed for classification of injury severity and estimation of mortality. The Injury Severity Score (ISS) for assessment of trauma severity summarizes the squares of the Abbreviated Injury Scores (AIS) of the 3 most affected body regions, and ranges from 0 to 75 points (0 - no injury to 75 non-survivable trauma) [7]. The AIS as defined by association for the advancement of automotive medicine (http://www.carcrash.org) was assessed and documented by two different qualified physicians (consultant trauma surgeon and anaesthesiologist) when the data was entered in the German Trauma Registry. For calculation of the New Injury Severity Score (NISS), the three highest AIS numbers are squared and added regardless of the body regions [8].The Revised Injury Severity Classification Score 2 (RISC2) was developed based on the data from the German Trauma Registry. Ten differently weighed indicators are used to estimate the chance of death, which is stated as a percentage [9]. The mentioned sores are all included in the Trauma Registry data set and automatically calculated based on the parameters entered in the registry.

### Statistical analysis

Normal distribution of the data was tested using the Shapiro-Wilks test. Continuous variables were expressed as median and interquartile range (IQR) (25th percentile, 75th percentile). Categorical variables were analysed using χ2 test. For continuous variables between-group differences the two tailed t-test or Mann-Whitney test was employed as appropriate. A two tailed test approach was chosen to detect a statistical difference indicative for a survival benefit of PHI vs. ETI and vice versa. In order to confirm or reject our null-hypothesis, the level of significance was set at *p* < 0.05. Statistical calculations were performed using GraphPad Prism 5.03 (GraphPad Software, La Jolla, CA, USA).

## Results

**Fig. 1:**
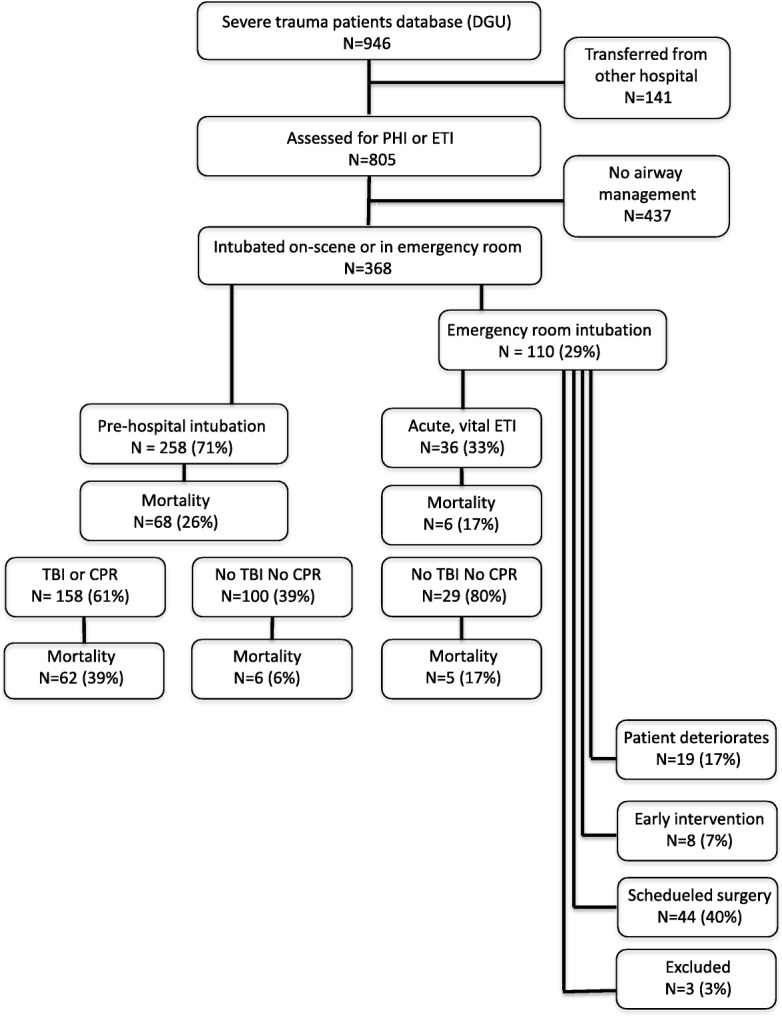
Flowchart of patient enrollment

A total of 946 patients were enrolled in the study (Fig. 1). Of the 368 patients who required advanced airway management, 258 (71%) were intubated during the pre-hospital phase (PHI) and 110 (29%) during the emergency room period (ERI). After categorizing ERI patients as described in Table 1, 36 patients (33%) matched the criteria for group 1, namely ERI due to acute vital indication. Therefore, a total of 294 patients met the study criteria and were subsequently analysed. Demographic data was comparable between groups (Table 2).

**Table 2:** Demographic data of the study population

		PHI	ERI	*p* value
	n	258	36	
Age	Median (IQR)	45,5 (24–60)	53 (36,3-65,8)	NS
Gender	Male	202 (78%)	24 (67%)	NS
	Female	56	12	NS
Mechanism of injury	MVA	143 (55%)	21 (58%)	
	Fall > 3 m	61 (24%)	8 (22%)	
	Fall < 3 m	17 (7%)	3 (8%)	
	Others	30 (12%)	3 (8%)	
	No information	7 (3%)	1 (3%)	

### Outcome and clinical data for all PHI and ETI patients

30-day mortality for the PHI group was 26.4% vs. 16.7% in the ERI group (*p* = 0.3). Additionally, there were no significant differences in lengths of intensive care unit (ICU) stay and mechanical ventilation between groups. Median RISC2 score, indicative for mortality, was significantly higher in the PHI group when compared with ERI patients (15.5 (4.2–71.1) % vs. 6.3 (1.2–23.7) %; *p* = 0.001). Median ISS and NISS scores were also higher in the PHI vs. ERI group, but not found significantly different with 33 (24–43) vs. 25 (20–38) points (*p* = 0.1), and 41 (27–57) vs. 38 (29–48) points (*p* = 0.6) (Table 3).

**Table 3:** Comparison between PHI and ERI patients

	Variable	PHI Number or Median (IQR)	ERI Number or Median (IQR)	*p* value
	n	258	36	
	Mortality	68 (26.4%)	6 (16.7%)	0.3
Pre-hospital	GCS	6 (3–10)	14 (12–15)	0.0001
	SBP	110 (80–130)	100 (90–117)	NS
	Heart rate	97 (75–116)	98 (85–180)	NS
	Respiratory rate	14 (10–18)	15 (12–20)	0,014
	SpO_2_	89 (80–95)	94 (90–98)	< 0.0001
	Cardiac arrest	30	0	0.0342
	Time on scene (min)	30 (21–40)	20 (15–28)	0.0002
	Transport time (min)	19 (13–26)	18 (10–21)	NS
	HEMS	194 (75%)	26 (72%)	NS
	Ground ambulance	64 (25%)	9 (28%)	NS
In-hospital	SBP	106 (80–125)	89 (70–145)	NS
	Heart rate	95 (80–160)	96 (82–115)	NS
	SpO_2_	98 (94–99)	92 (83.5–96)	< 0.0001
	PaO_2_	204 (94,5–317)	146 (64.5–195)	0.0061
	PaCO_2_	43.6 (37.2–50.5)	43.8 (38.7–50.9)	NS
	BE	−3.95 (−7 - -1,7)	−4.35 (−8,4 - -1,83)	NS
	Body temperature	35.6 (34.6–36.1)	36 (35–36.4)	NS
	Time to CT (min)	13 (9–19)	17 (10–23.5)	NS
	ICU stay (h)	312 (72–528)	76 (144–450)	NS
	Mech. ventilation (h)	144 (24–336)	120 (24–324)	NS
Scores	ISS	33 (24–43)	25 (20–38)	NS
	NISS	41 (27–57)	38 (29–48)	NS
	RISC2	15.5 (4,2-71,1)	6.3 (1,2-23,7)	0.0013

Significant differences were noted in the median initial oxygen saturation (SaO_2_) assessed on scene, which was lower in the PHI vs. ERI group (89.5% (80–95) vs. 94%(90–98); *p* < 0.0001). Thirty PHI patients required cardiopulmonary resuscitation (CPR). One hundred twenty-eight PHI patients suffered from traumatic brain injury, which resulted in higher median (IQR) AIS values for the head in PHI vs. ERI patients (4 (2–5) vs. 0 (0–3) points; *p* = 0.0001), and lower median GCS values (6 (3–10) vs. 14 (12–15; (*p* = 0.0001).

First available median (IQR) SpO_2_ and arterial oxygen pressure (PaO_2_) values after admission were significantly higher in the PHI group when compared with the ERI group (98 (94–99)% vs. 92 (83–96)%, (*p* < 0.0001); and 204 (94–317) mmHg vs. 146 (64–195) mmHg (*p* = 0.0061), respectively).

The differences in systolic blood pressure (SBP), heart rate, arterial carbon dioxide pressure (PaCO_2_), base excess and body temperature were not significant between groups upon hospital admission. However, after excluding all cardiac arrest cases, 57 / 228 PHI patients (25%) required vasopressor drugs for hemodynamic stabilization after induction of emergency anaesthesia in the pre-hospital phase whereas only 2 / 36 (6%) ERI patients needed vasopressor support in the ER (*p* = .002).

To test the hypothesis that decision making for PHI might be influenced by the expected transport time, and might thus eventually be postponed when the hospital is nearby, we compared median (IQR) transport times for PHI and ERI groups. No significant difference was observed in PHI 19 (13–26) minutes vs. ERI 18 (10–21) minutes (*p* = 0.110). Time on scene of the accident, however, was significantly longer in PHI group with a median of 30 (21–40) minutes vs. ERI group with a median of 20 (15–28) minutes (*p* = 0.0002). The median time span between hospital admission and start of computer tomography (CT) diagnostics was faster in the PHI group with 13 (9–19) minutes vs. 17 (10–23,5) minutes in the ERI group (*p* = 0.05).

### Outcome after exclusion of severe head injury and/or cardiac arrest

After exclusion of severe traumatic brain injury (TBI), defined by a head ISS of 4 points or more and of patients requiring cardio-pulmonary resuscitation (CPR), we noted a trend towards a lower mortality rate in PHI vs. ETI patients (6/100 vs. 5/29; *p* = 0.07). Nonetheless, the difference in initial GCS, on-scene and in-hospital SaO_2_, as well as in-hospital PaO_2_ values between groups was still significant (Table 4).

**Table 4:** Comparison between groups after excluding TBI and CPR patients

	Variable	PHI Number or median (IQR)	ERI Number or median (IQR)	*p* value
	n	100	29	
	Mortality	6 (6%)	5 (17.2%)	0.07
Pre-hospital	GCS	10 (6–14,5)	15 (13–15)	< 0.001
	SBP	100 (80–125)	99 (90–110)	NS
	Heart rate	100 (90–116)	99 (85–120)	NS
	Respiratory rate	15 (12–20)	16 (12–20)	NS
	SpO_2_	89.5 (83–96)	94 (90–98)	0,03
In-hospital	SBP	99.5 (80–120)	81 (70–126)	NS
	SpO_2_	98.5 (95–99)	92 (84–96)	< 0.0001
	PaO_2_	192.5 (92.8–311.5)	112 (63–195)	< 0.0001
	PaCO_2_	42 (36.9–50.5)	43.6 (38.1–52.5)	NS
	BE	−4.4 (−2 - -6.5)	−4.6 (−1.9 - -8.3)	NS
Scores	ISS	25 (18–36)	29 (19–38)	NS
	NISS	29 (22–41)	36 (28–45,5)	0.0479
	RISC2	6.0 (1.2–14.8)	3.6 (1.1–9.5)	NS

### Outcome of awake, hypotensive trauma patients

A total of 28 patients with a GCS of ≥12 and a SBP of ≤90 mmHg at the accident scene who had to be intubated during the course of pre-hospital or emergency room treatment were admitted to the Salzburg Trauma Centre during the study period. All patients survived when pre-hospital emergency anaesthesia was induced (*N* = 14) or postponed until ER admission (N = 14).

## Discussion

We sought to contribute to the discussion about the risk-benefit ratio of emergency anaesthesia and intubation by retrospectively evaluating a subgroup of severe, but spontaneously breathing trauma patients. Interestingly, we found no significant difference in mortality rates between patients who underwent pre-hospital intubation and those who were intubated immediately after emergency room admission. Nonetheless, there was a trend towards lower mortality in patients who underwent airway management on scene. Furthermore, there was no difference in the length of mechanical ventilation or ICU stay. Pre-hospital intubation prolonged the time spent on-scene by an average of 10 min, while intubation in the emergency room delayed the time until start of computer tomographic trauma imaging by an average of 4 min.

There is no doubt that insufficient or absent breathing, as well as severe head injury with impaired respiration require immediate, time critical airway management. After exclusion of head injured and cardiac arrest patients, the clinical findings and outcome parameters observed in our study were not different between groups, thus suggesting that pre-hospital intubation does not influence mortality. The equal mortality rates between pre-hospital and emergency room intubation, correspond with the results of comparable studies. Crewdson et al. found that pre-hospital vs. in-hospital emergency anaesthesia and intubation was associated with a 3-fold higher mortality in awake hypotensive trauma patients [5]. Bochicchio et al. reported a significantly increased mortality and morbidity in trauma patients without severe head injury who were intubated in the field compared to those who were intubated immediately after hospital admission [10]. A systematic review and meta-analysis conducted by Fevang et al. indicated that only one of 21 studies was able to show a positive effect of pre-hospital intubation, whereas 12 publications reported higher mortality rates [6]. Considering the complex pathophysiology after trauma and the current literature, we choose the statistical approach of defining the null-hypothesis that survival rates are comparable between both groups. Since the observed difference in survival rates did not reach statistical significance, the null-hypothesis was not rejected and we found neither harm nor benefit of PHI vs. ETI. Our results do not open for an alternative hypothesis that mortality rate could be different between both groups.

Our findings of decreased initial GCS, respiratory rate, oxygen saturation and increased RISC2 scores in the PHI group can be explained by a high number of severe head injuries, as well as pre-hospital cardiac arrests in this cohort. Several studies have shown that patients suffering of severe traumatic brain injury benefit from early intubation and artificial ventilation, provided that extreme hypo- and hyperventilation are avoided [2, 11]. Furthermore, guidelines for the treatment of (traumatic) cardiac arrest call for airway management through emergent endotracheal intubation [12]. Thus, after exclusion of all cases with severe traumatic brain injury and/or pre-hospital cardiac arrest, data comparison between PHI and ERI groups showed that the differences in GCS and initial oxygen saturation were still apparent, while respiratory rates and RISC2 scores were not significantly different. The approximation of RISC2 scores can be explained by the relatively high weighing of head injuries in the calculation of the score. We did not observe a significant difference in mortality in this subgroup, which is in accordance with the aforementioned studies of trauma patients without severe head injury [5].

The fact that mortality rates were comparable with a non-significant trend towards lower rates in PHI patients (6 vs. 17%) might indicate that the emergency medical physicians involved in our system are experienced anaesthesiologists and thus well trained in patient selection for and performance of intubation and emergency anaesthesia. Moreover, EMS physicians might have been able to identify and manage patients eligible for transport without securing the airway in advance. Accordingly, a higher number of cases would be needed for more valid data analysis.

When looking at logistics and length of time until hospital arrival, we found a significantly increased on scene time for the PHI group, which corresponds with the findings of Hussmann et al., who conducted a matched-pairs analysis on pre-hospital intubation in 2011 [13]. The delay until emergency room treatment and diagnostics for the PHI group does not seem to affect overall mortality. This is in accordance with the findings of Newgard et al., who conducted an analysis on a similar patient cohort, which showed an increase in mortality associated with a pre-hospital phase of > 60 min only in patients requiring early critical resources [14].

Despite the fact that hemodynamic variables upon ER admission were comparable between groups, it has to be pointed out that a significantly higher percentage of patients in the PHI group received vasopressor drugs when compared with the ERI group. This finding sheds light on the marked hemodynamic effects of anaesthetic drugs, combined with a more difficult management of general anaesthesia in the pre-hospital setting [15, 16].

Our findings of a non-significant difference in mortality associated with pre-hospital emergency anaesthesia in a subgroup of patients who were awake (GCS ≥13), yet hypotensive and presumably in shock (SBP ≤90 mmHg) on scene corresponds with the results of similar studies. As such, Crewdson et al. conducted an investigation in 2018 and found a significant increase in mortality when pre-hospital emergency anaesthesia was performed on awake, hypotensive trauma patients [5]. Although we were not able to confirm this survival benefit, we speculate that airway management in awake hypovolemic shock patients can be postponed safely until hospital admission. We previously commented on the study of Crewdson et al. regarding our findings on the topic [17].

Concerning the indications for pre-hospital anaesthesia and intubation, Rognas et al. published a prospective observational study on the topic in 2013. They found that a decreased level of consciousness was the most common indication for considering pre-hospital intubation among emergency physicians, followed by hypoxemia and ineffective ventilation [18]. These findings seem to go along with our data, which show a significantly decreased GCS and respiratory rate, as well as decreased oxygen saturation on scene for the PHI group vs. the ERI group.

Some significant limitations of this study must be noted. First, it is noteworthy that the PHI group also comprised patients with insufficient respiration and physicians deemed transport without airway control as irresponsible. In contrast, patients intubated in the ER were considered to survive transport at least until hospital admission. Thus the comparable outcome data might in part be explained by the competence of our EMS physicians. Second, the significantly higher RISC2 scores and significantly lower Glasgow Coma Scores of PHI cases presumably trace back to a higher number of severe head injuries, as well as cardiac arrests in this group. We sought to eliminate these factors by excluding patients with a head ISS > 3 and/or pre-hospital cardiopulmonary resuscitation (CPR) in a subgroup analysis. The number of patients with severe head injuries in the ERI group was too low for sufficient data analysis of this subgroup. Third, our data derives from the Trauma Registry, the electronic patient data management and hospital documentation system, as well as from hand-written emergency physician records. The latter frequently lack information or provide data of questionable quality, which could be explained by the nature of the pre-hospital circumstances with limited time for documentation during emergency care. As in every retrospective study, the quality of data has a significant impact on the findings and interpretation. Finally, due to the retrospective character of the study no power analysis for controlling study population size was performed, but the number of patients included in this trial was comparable or even higher than in published literature. It might be possible that some subgroup analysis might lack power, wherefore further studies in this important field are needed.

## Conclusions

There was no statistical difference in mortality rates of spontaneously breathing trauma patients intubated on-scene when compared with patients intubated immediately after hospital admission. Due to the retrospective study design and small case number, further studies evaluating the impact of airway management timing in spontaneously breathing trauma patients are warranted.

## Data Availability

The datasets generated during and/or analysed during the current study are available from the corresponding author on reasonable request.

## Abbreviations

AIS: Abbreviated Injury Scores
CPR: Cardiopulmonary Resuscitation
CT: Computer Tomography
EMS: Emergency Medical Service
ER: Emergency Room
ERI: Emergency Room Intubation
GCS: Glasgow Coma Scale
HEMS: Helicopter Emergency Medical Service
ICU: Intensive Care Unit
IQR: Interquartile Range
ISS: Injury Severity Score
NISS: New Injury Severity Score
PaCO_2_: Arterial Carbon Dioxide Pressure
PACS: Picture Archiving and Communication System
PaO_2_: Arterial Oxygen Pressure
PDMS: Patient Data Management System
PHI: Pre-Hospital Intubation
RISC: Revised Injury Severity Classification
SaO_2_: Arterial Oxygen Saturation
SBP: Systolic Blood Pressure
TBI: Traumatic Brain Injury
